# Interferon gamma induces inflammatory responses through the interaction of CEACAM1 and PI3K in airway epithelial cells

**DOI:** 10.1186/s12967-019-1894-3

**Published:** 2019-05-09

**Authors:** Yichun Zhu, Dongli Song, Yuanlin Song, Xiangdong Wang

**Affiliations:** 1 Zhongshan Hospital Institute of Clinical Science, Shanghai, China; 2 Department of Respiratory Medicine of Zhongshan Hospital, Fudan University, Shanghai, China; 3 Shanghai Institute of Clinical Bioinformatics, Shanghai, China; 4 Shanghai Engineering Research for AI Technology for Cardiopulmonary Diseases, Shanghai, China

**Keywords:** COPD, Lung cancer, CEACAM1, PI3K/Akt pathway, IFN-γ

## Abstract

**Background:**

Interferon gamma (IFNγ) plays an important role in the development of chronic lung diseases via the production of inflammatory mediators, although the exact mechanism remains unclear. The present study aimed at investigating the potential mechanisms by which IFNγ induced over-production of interleukins through the interaction between carcinoembryonic antigen-related cell adhesion molecule 1 (CEACAM1) and phosphatidylinositol-4,5-bisphosphate 3-kinase (PI3K) pathway.

**Methods:**

IFN-γ induced over-production of interleukin (IL) 6 and IL8, and RNA expression of CEACAM1 and its subtypes or PI3K and its subtypes in human bronchial epithelial cells (HBE). The production of IL6 and IL8 or cell proliferation and movement were also evaluated in cell^CEACAM1−^ or cell^CEACAM1+^ after the induction of IFN-γ. Roles of PI3K subtype proteins, e.g. PI3Kp110α/δ, Akt, p110α/γ/δ/β/mTOR, PI3Kp110α/δ/β, PI3Kp110δ, or pan-PI3K in IFN-γ-induced CEACAM1 subtype alterations were furthermore validated using those proteins of PI3K subtypes.

**Results:**

CEACAM1, especially CEACAM1-S isoforms, was significantly up-regulated in HBE cells after treatment with IFN-γ. CEACAM1 played roles in expression of IL-6 and IL-8, and facilitated cellular proliferation and migration. IFN-γ up-regulated the expression of CEACAM1 in airway epithelial cells, especially CEACAM1-S isoforms, promoting cellular proliferation, migration, and the production of inflammatory factors. PI3K (p110δ)/Akt/mTOR pathway was involved in the process of IFN-γ-upregulated CEACAM1, especially CEACAM1-S. On the other hand, CEACAM1 could promote the activation of PI3K/Akt/mTOR pathway.

**Conclusion:**

IFN-γ could induce inflammatory responses, cellular growth and proliferation through the interaction of CEACAM1 (especially CEACAM1-S isoforms) and PI3K(p110δ)/Akt/mTOR in airway epithelial cells, which might be new alternative of future therapies against epithelial transition from inflammation to cancer.

**Electronic supplementary material:**

The online version of this article (10.1186/s12967-019-1894-3) contains supplementary material, which is available to authorized users.

## Background

Carcinoembryonic antigen-related cell adhesion molecule 1 (CEACAM1) belongs to immunoglobulin superfamily, presents in various cells, and participates in cellular proliferation, apoptosis, angiogenesis, tumor metastasis, and pathogen recognition. CEACAM1 mediates transcellular transcytosis, suppresses immune cell activities and inflammatory responses, and plays a critical role in the development of lung cancer and acute exacerbation of chronic obstructive pulmonary diseases (AECOPD). We emphasized the importance to understand the interaction and network of driver molecules and develop new biomarkers and therapeutics during the transformation from chronic lung inflammation to cancer [1]. There is a clear correlation between COPD and lung cancer, although molecular mechanisms of COPD-lung cancer transits remain unclear. The chronic inflammation in COPD might be associated with increased DNA errors during tissue remodeling after injury, while acute exacerbations of COPD might worsen the condition, and finally resulting in amplification of carcinogenic effects [2–7]. CEACAM1 can be one of potential target candidates to play a critical role in the COPD-lung cancer transits. The human CEACAM1 gene has 12 different isoforms via different splicing, of which isoforms (4L, 3L, 4S, 3S, 3, 4C1, 3C2) are involved in immune regulation or inflammation [8]. Lipopolysaccharide (LPS) and interferon gamma (IFN-γ) as key intermediates contribute to the initiation of inflammation probably through signal pathways of PI3K and NF-κB [9, 10]. PI3K activation plays a central role in the development of airway inflammation and tissue remodeling [11]. CEACAM1 was also a key factor in inflammation, fibrosis and tissue remodeling [12, 13]. PI3K inhibitor could induce apoptosis in high CEACAM1 expressed cells, rather than CEACAM1 knockdown cells [14]. These studies suggested that the regulations of PI3K signaling on inflammation, tissue remodeling and apoptosis might more dependent on CEACAM1.

We previously evaluated the dynamic gene expression profiling of peripheral blood mononuclear cells (PBMCs) from patients with AECOPD on day 1, 3 and 10 after hospitalization, and selected up-regulated CEACAM1 as one of AECOPD-specific biomarkers [15]. The role of CEACAM1 on regulation of adhesion and function of B cells is in a PI3K-dependent manner [16–18]. IFN-γ was found to induce the malignant transformation of healthy lactating bovine mammary epithelial cells, closely associated with the promotion of cell growth and even the carcinogenesis of breast cancer [19]. The present study aims at evaluating the expression of CEACAM1 in lung diseases and the profiling of CEACAM1 isoforms and PI3K subtypes after IFN-γ treatment, and investigating the role of PI3K pathway in IFN-γ-induced CEACAM1 expression in airway epithelial cells. In order to determine the role of CEACAM1 in alternations of IFN-γ-induced cell function, gene expression of inflammatory factors and proliferation and migration of cell^CEACAM1−^ were furthermore determined.

## Materials and methods

### Cell lines and reagents

Human bronchial epithelial cell line HBE cells were cultured in RPMI 1640 supplemented with penicillin (100 U/ml), streptomycin (100 mg/ml), and 10% heat inactivated fetal bovine serum (FBS). Lipopolysaccharide(LPS, Escherichia coli, 055:B5)was purchased from Sigma (Missouri, USA). Interferon-γ was purchased from R&D Systems (Minnesota, USA). PI3K/Akt/mTOR inhibitors (GDC0941, GSK690693, BEZ235, LY294002 and CAL101) were purchased from Biovision (California, USA). SHBM1009 (a new PI3K/mTOR inhibitor) was synthesized by Fudan University. CEACAM1 and GAPDH antibodies for western blot were purchased from Abcam (Hong Kong, China). SYBR Premix Ex Taq was from TaKaRa (Shiga, Japan). Lipofectamine™ 2000 Transfection Reagent was from Invitrogen (Grand Island, NY, USA).

### Small interfering RNA transfection

Small interfering RNA (siRNA) transfections were performed, according to the manufacturer’s protocol, in 6-well plates or 24-well plates, using Lipofectamine™ 2000 with siRNA duplexes (Table 1) targeting CEACAM1 and a double-stranded RNA negative control (GenePharma, Shanghai, China). As for 6-well plates, 4 μl of Lipofectamine™ 2000 and 160 pmol of each siRNA were transfected; as for 24-well plates, 1 μl of Lipofectamine™ 2000 and 40 pmol of each siRNA were transfected, each group in triplicate. Cells were then treated with IFN-γ for an additional 24 h and then prepared for quantitative RT-PCR, western blot, and other measurements 24 h after transfection.

**Table 1:** Sequences mentioned in the article

Name	Sequences	
	Forward primer	Reverse primer
CEACAM1	5′-GCTGGCATTGTGATTGGAGTA-3′	5′-TTAGGTGGGTCATTGGAGTG-3′
CEACAM1-4S	5′-AAGACGATCATAGTCACTGAGCT-3′	5′-ATTGGAGTGGTCCTGAGCTG-3′
CEACAM1-4L	5′-AAGACGATCATAGTCACTGAGCT-3′	5′-GGAGACTGAGGGTTTGTGCT-3′
CEACAM1-3S	5′-TCATAGTCACTGATAATGCTCTACC-3′	5′-ATTGGAGTGGTCCTGAGCTG-3′
CEACAM1-3L	5′-TCATAGTCACTGATAATGCTCTACC-3′	5′-GGAGACTGAGGGTTTGTGCT-3′
CEACAM1-4C1	5′-AAGACGATCATAGTCACTGAGCT-3′	5′-TTGCACACCATTGACAGAGT-3′
CEACAM1-3	5′-CAGTGACCCAGTCACCTTGA-3′	5′-TGGACTTGTTTGTGCCTGTTG-3′
CEACAM1-3C2	5′-CAAGACGATCATAGTCACTGAGTC-3′	5′-AGAGGGACATATAGGAAGGGGT-3′
PIK3CA	5′-GGGATGATTTACGGCAAGATA-3′	5′-CCACACAGTCACCGATTGA-3′
PIK3CB	5′-TGCGACAGATGAGTGATGAA-3′	5′-TCCTCCGATTACCAAGTGCT-3′
PIK3CD	5′-GCCAACATCCAACTCAACAA-3′	5′-CCACACAATAGCCAGCACAG-3′
PIK3CG	5′-TAGACCACCGTTTCCTCCTG-3′	5′-CGTGAGTTTGTCAGCATTGA-3′
PIK3C2A	5′-GATTACCTGGGCCTTCCAC-3′	5′-AGTGGGCATTCTTGGATTGA-3′
PIK3C2B	5′-TCCACCTTGAACTACCTCGTC-3′	5′-AAGTCTCCATCAGCCAGCAG-3′
PIK3C2G	5′-CTCCTGGCATCAAGTTAGCA-3′	5′-TCTGGAATCATCAGCACCAT-3′
PIK3C3	5′-ATCCCGTTGCCTTTAGAACC-3′	5′-TGCCTCCATCTTCCGTCTTA-3′
PIK3R1	5′-GGACGGCGAAGTAAAGCAT-3′	5′-TGACATTGAGGGAGTCGTTG-3′
PIK3R2	5′-GATGGGCACTATGGCTTCTC-3′	5′-TGCTGGTATTTGGACACAGG-3′
PIK3R3	5′-AGCACAACGACTCCCTCAAC-3′	5′-AAATGCCAGAGAACCACCTC-3′
PIK3R4	5′-TGGCATTTGTGTCCCTTTGT-3′	5′-TGCTGGATGAGTTGCTGAAG-3′
PIK3R5	5′-TACACCACACTTCCCACCAG-3′	5′-CTCAGCCCTCACCAGTCTCT-3′
PIK3R6	5′-TTCCCACTTCTCCCACTGTC-3′	5′-TGCTGTTCCTTGCTTCCAAT-3′
IL-6	5′-GACAGCCACTCACCTCTTCAG-3′	5′-CATCCATCTTTTTCAGCCATC-3′
IL-8	5′-TTGCCAAGGAGTGCTAAAGAA-3′	5′-GCCCTCTTCAAAAACTTCTCC-3′
MCP-1	5′-AGGAACCGAGAGGCTGAGA-3′	5′-GGAATGAAGGTGGCTGCTAT-3′
TGF-β	5′-GCCAGAGTGGTTATCTTTTGATG-3′	5′-AGTGTGTTATCCCTGCTGTCAC-3′
VEGF	5′-AGGGCAGAATCATCACGAAGT-3′	5′-AGGGTCTCGATTGGATGGCA-3′
GAPDH	5′-CCACCCATGGCAAATTCCATGGCA-3′	5′-TCTACACGGCAGGTCAGGTCCACC-3′
siRNA-CEACAM1	5′-CACCUUGAAUGUCACCUAUTT-3′	5′-AUAGGUGACAUUCAAGGUGTT-3′
siRNA-NC	5′-UUCUCCGAACGUGUCACGUTT-3′	5′-ACGUGACACGUUCGGAGAATT-3′

siRNA-NC is a negative control siRNA with the same nucleotide composition but which lacks significant sequence homology to CEACAM1

### Measurement of gene expression

Total RNA was isolated using a guanidinium isothiocyanate/chloroform based technique (TRIZOL, Invitrogen, USA) and measured with OD 260 nm. RNA was subsequently reversed and transcribed to cDNA by using PrimeScript RT reagent Kit (TaKaRa, China). Real-time PCR was carried out using a CFX96 Touch Real-time PCR instrument (Bio-Rad, USA) with the two-stage program parameters, as follows: 1 min at 95 °C, and then 40 cycles of 5 s at 95 °C and 30 s at 60 °C. The sequences of the primer sets used for this analysis are listed in Table 1. Specificity of the produced amplification product was confirmed by examination of dissociation reaction plots. Each sample was tested in triplicate with quantitative RT-PCR, and each group had six wells.

### Western blot

Protein samples were mixed with one-fourth volume of SDS sample buffer, boiled for 5 min, and then separated through 10% SDS-PAGE gels. After electrophoresis, proteins were transferred to PVDF membranes through electrophoretic transfer. Membranes were blocked in 5% bovine serum albumin for 2 h, rinsed and incubated with primary antibodies directed against CEACAM1 (rabbit monoclonal, 1:5000; Abcam) or GAPDH (mouse monoclonal, 1:1000; Beyotime) at 4 °C overnight, then washed in TBS-tween thrice and treated with secondary HRP-conjugated anti-rabbit (1:1000) or anti-mouse (1:1000) for 2 h at room temperature. Secondary antibody-bound protein was detected using the ECL kit (Beyotime). Data were analyzed using the Image J software.

### Wound-healing assay

HBE cells transfected with siRNA-CEACAM1 or siRNA-NC for 24 h were then treated with IFN-γ in 24-well plates. After another 24 h, a sterilized pin was used to wipe off the adherent cells on two intersecting lines, to create the wounds in each well. The migration of HBE was assessed using an inverted light microscope at the original magnification 40× and migrated distances of HBE were measured at 0, 12, 24, 48 or 72 h.

### Cell proliferation assay

The effect of CEACAM1 knockdown on HBE cell proliferation was detected by Cell counting kit-8 (CCK-8, Beyotime, Shanghai, China). HBE cells transfected with siRNA-CEACAM1 or siRNA-NC were plated to 96-well plates. After IFN-γ treatment for 0, 24, 48, 72 h, CCK-8 was added (10 μl) to each well, and incubated in 37 °C. The absorbance at 450 nm was measured 2 h later. Each group has six wells.

### Statistical analysis

Data was presented as mean ± standard errors. Statistical analysis was performed using SPSS software (SPSS 20.0; SPSS Inc; Chicago, IL, USA). Data were evaluated using ANOVA with LSD test for multiple comparisons and Students *t* test between two groups. Increased rates of total cell number and differentiation were calculated as the following: Rate (%) = (value at each time point-value of primary seeding cells)/value of primary seeding cells × 100. P-values less than 0.05 were considered to be statistically significant.

## Results

**Fig. 1:**
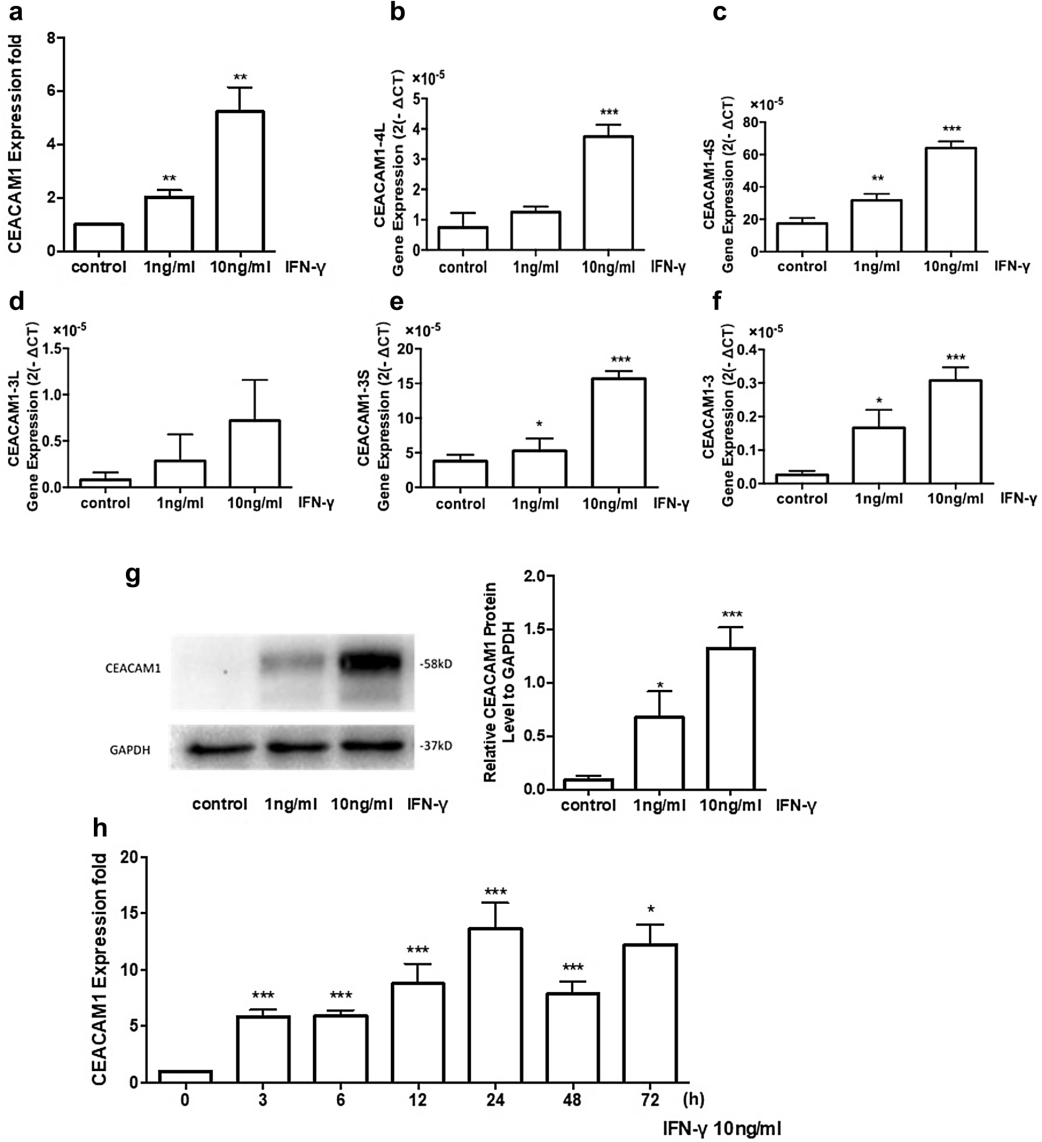
Expression of CEACAM1 after IFN-γ stimulation in HBE cells. mRNA expression of CEACAM1 (**a**), CEACAM1-4L (**b**), CEACAM1-4S (**c**), CEACAM1-3L (**d**), CEACAM1-3S (**e**), CEACAM1-3 (**f**), as well as protein levels of CEACAM1 protein (**g**) were measured in HBE cells treated with vehicle (control) or with IFN-γ at concentration of 0, 1, 10 ng/ml for 24 h. Dynamic expression of CEACAM1 gene in HBE cells 0, 3, 6, 12, 24, 48, 72 h after treatment with vehicle (control) or with IFN-γ at 10 ng/ml (**h**). *, **, ***p-values less than 0.05, 0.01 and 0.005, as compared to control

**Fig. 2:**
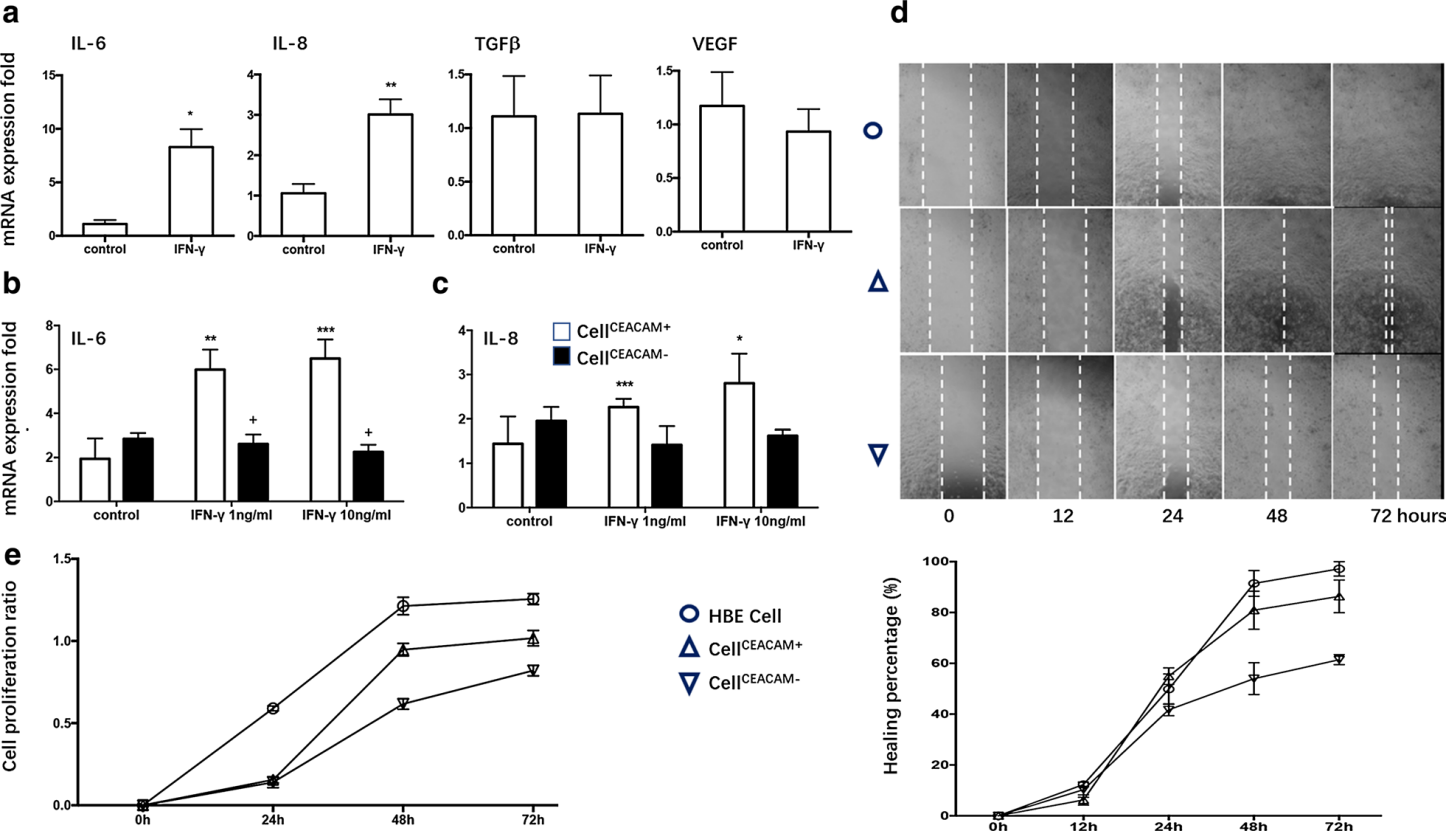
Role of CEACAM1 in HBE cells with IFN-γ treatment. mRNA expression of IL-6, IL-8, TGFβ, and VEGF in HBE cells treated with vehicle (control) or with IFN-γ at 10 ng/ml for 24 h (**a**). mRNA expression of IL6 (**b**) and IL8 (**c**) in cell^CEACAM1−^ or cell^CEACAM1+^ treated with vehicle (control) or with IFN-γ at 1.0 or 10 ng/ml. Cell movement measured by wound healing assay (**d**) and cell proliferation measured by CCK8 0, 12, 24, 48, or 72 h after treatment with vehicle (HBE cell) and treated with IFN-γ. Healing percentage (%) = (S_0_ − S_t_)/S_0_ × 100% (S_0_: wound area at 0 h; S_t_: wound area at specific time point). Cell proliferation rate = (OD_t_-OD_0_)/OD_0_ (OD_0_: OD value at 450 nm at 0 h after treated with IFN-γ; OD_t_: OD value at 450 nm at t hours after treated with IFN-γ). *, **, ***p-values less than 0.05, 0.01 and 0.005, as compared to control

We evaluated mRNA expression of CEACAM1 in HBE cells stimulated by LPS or IFN-γ, respectively, and found no significant difference of CEACAM1 expression 24 h after LPS stimulation at the concentration of 0.1 μg/ml or 1 μg/ml (Additional file 1: Figure S1), while mRNA (Fig. 1a) and protein (Fig. 1g) expression of CEACAM1 significantly up-regulated after IFN-γ stimulation at 1 or 10 ng/ml in a concentration-dependent pattern. CEACAM1 mRNA expression significantly increased from 3 h and on after IFN-γ stimulation at 10 ng/ml and reached the peak at 24 h (Fig. 1h). Of CEACAM-1 subtypes, CEACAM1-4S and -3S isoforms are dominant in HBE cells, and mRNA of CEACAM1-4S, 4L, 3S, and 3 isoforms increased significantly after IFN-γ stimulation (Fig. 1b–f). Of interleukin (IL)-6, IL-8, transforming growth factor-β (TGF-β), vascular endothelial growth factor (VEGF), and monocyte chemoattractant protein-1 (MCP-1), we found that IFN-γ stimulation increased expression of IL-6 and IL-8 in HBE cells (Fig. 2a), while not in cell^CEACAM1−^ (Fig. 2b, c). Delayed cell migration (Fig. 2d) and decreased cell proliferation (Fig. 2e) were noticed in cell^CEACAM1−^, as compared with HBE cells or cell^CEACAM1+^, respectively.

**Fig. 3:**
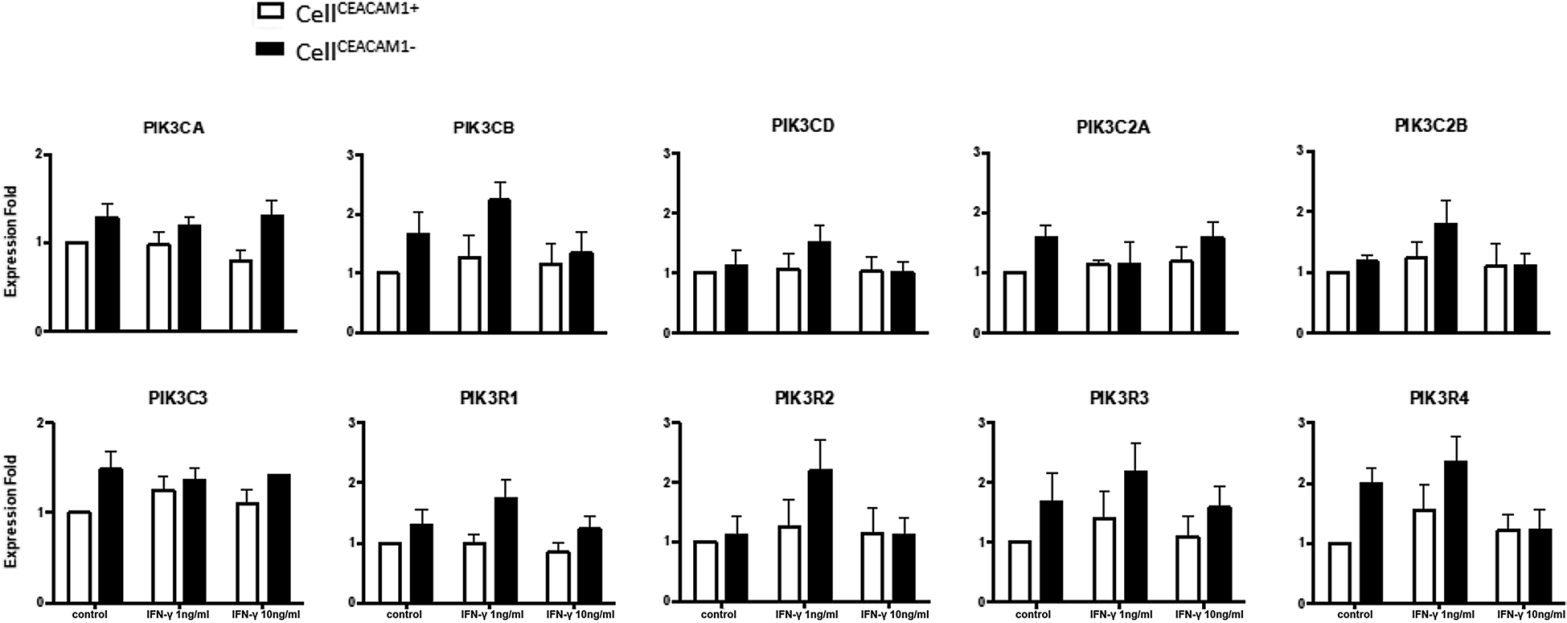
Effect of CEACAM1 on PI3K subtype gene expression. mRNA expression of 10 PI3K subunits (PIK3CA, PIK3CB, PIK3CD, PIK3C2A, PIK3C2B, PIK3C3, PIK3R1, PIK3R2, PIK3R3, PIK3R4) in cell^CEACAM1−^ or cell^CEACAM1+^ treated with vehicle (control) or with IFN-γ at 1.0 or 10 ng/ml. mRNA levels of PIK3CG, PIK3C2G, PIK3R5, PIK3R6 expression could not be detected

To define roles of CEACAM1 in PI3Ks, we evaluated the mRNA expression profiling of ten PI3K subtypes between HBE cell^CEACAM1+^ and cell^CEACAM1−^, and found the expression of PIK3CA, PIK3CB, PIK3C2A, PIK3C3, PIK3R3, and PIK3R4 up-regulated in cell^CEACAM1−^, as compared to those in cell^CEACAM1+^ (Fig. 3). The low concentration (1 ng/ml) of IFNγ increased the expression of PIK3CB, PIK3C2B, PIK3R1, and PIK3R2, but not the high concentration (10 ng/ml). This indicates a clear correlation of biological functions between CEACAM1 and PI3K subunits and the regulatory role of CEACAM1 in expression of PI3K subunits, of which some were regulated by the certain concentration of IFNγ. However, all these results were without statistical significance.

**Fig. 4:**
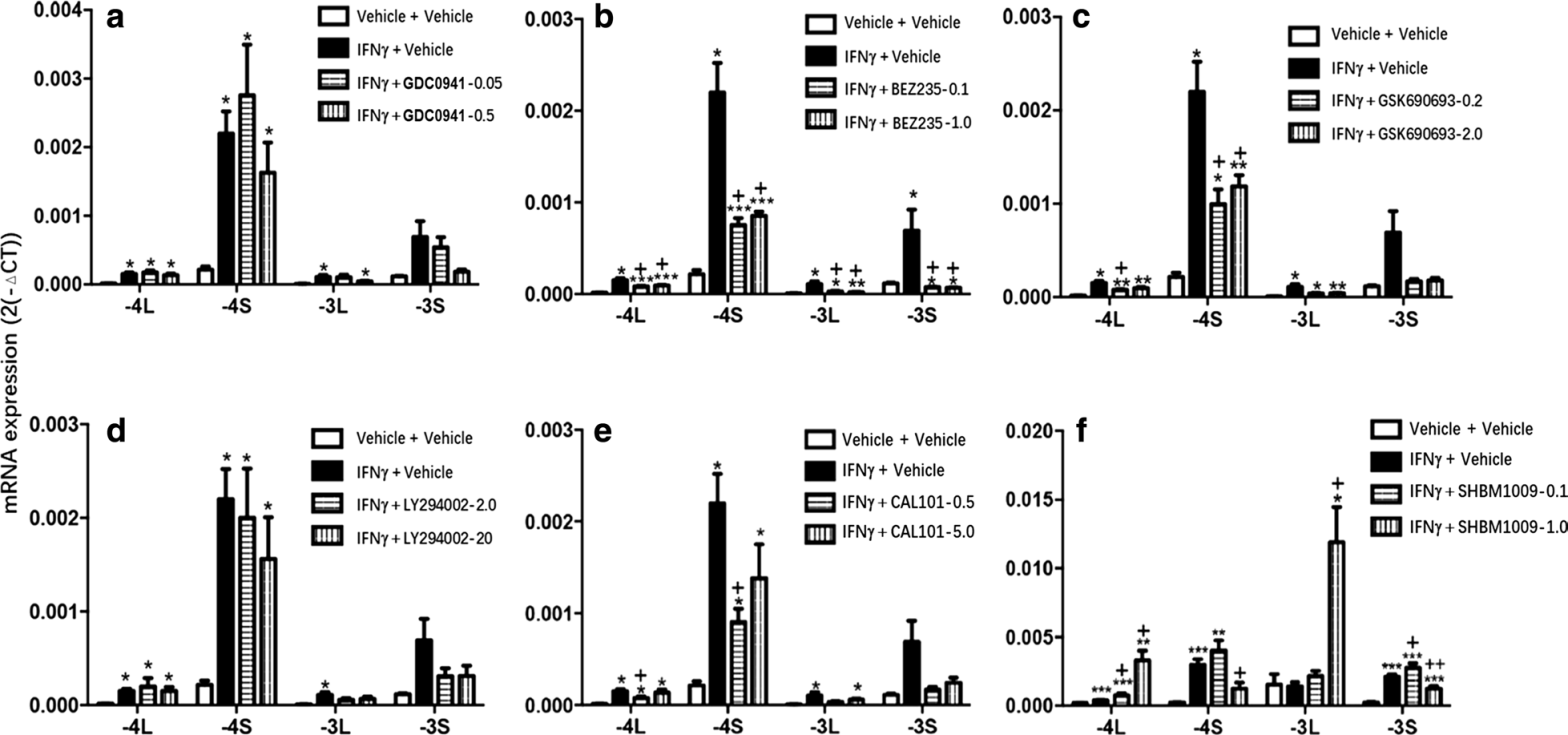
Effect of PI3K/Akt pathway inhibitors on IFN-γ induced CEACAM1 and its isoform expression. mRNA expression of CEACAM1-4L, -4S, -3L, and -3L in HBE cells treated with p110α/δ inhibitor (GDC0941) at 0.05 or 0.5 ng/ml (**a**), Akt inhibitor (GSK690693) at 0.1 or 1.0 ng/ml (**b**), p110α/γ/δ/β/mTOR inhibitor (BEZ235) at 0.2 or 2.0 ng/ml (**c**), p110α/δ/β inhibitor (LY294002) at 2.0 or 20 ng/ml (**d**), p110δ inhibitor (CAL101) at 0.5 or 5.0 ng/ml (E), pan-PI3K inhibitor (SHBM1009) at 0.1 or 1.0 ng/ml (F) 24 h after stimulation and treatment with vehicle (Vehicle + Vehicle), IFN-γ with vehicle, IFN-γ with various inhibitors. *, **, ***p-values less than 0.05, 0.01 and 0.005, as compared to HBE control; ^+, ++, +++^p-values less than 0.05, 0.01 and 0.005, as compared to IFN-γ with vehicle, respectively

**Fig. 5:**
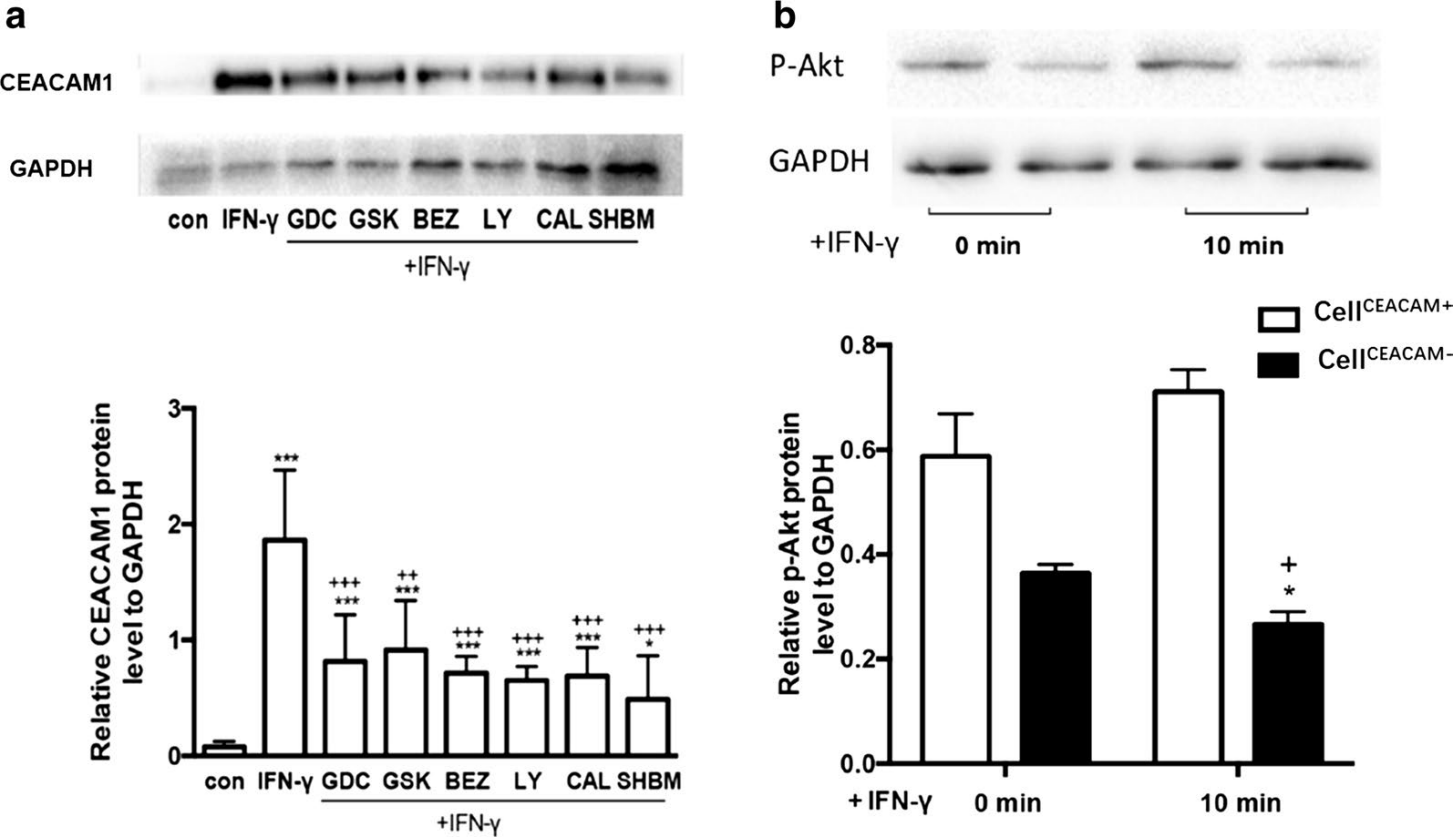
Interaction between CEACAM1 and PI3K. **a** Protein levels of CEACAM1 in HBE cells pretreated with GDC0941 (GDC), BEZ235 (BEZ), GSK690693 (GSK), LY294002 (LY), CAL101 (CAL), and SHBM1009 (SHBM) 24 h after stimulation with IFN-γ for 24 h, as compared with control group with vehicle (CON). *, **, ***p-values less than 0.05, 0.01 and 0.005, as compared to HBE control, or ^+, ++, +++^p-values less than 0.05, 0.01 and 0.005, as compared to IFN-γ with vehicle, respectively. **b** Levels of phosphorylated Akt protein (p-AKT) in cell^CEACAM1−^ or cell^CEACAM1+^ treated with vehicle (control) or with IFN-γ at 10 ng/ml for 0 and 10 min. *p-values less than 0.05, as compared to control, ^+^p-values less than 0.05, as compared to 0 min group, respectively

HBE cells were pretreated with different PI3K inhibitors, including GDC0941, GSK690693, BEZ235, LY294002, CAL101 and SHBM1009, and then stimulated by IFN-γ for 24 h. Of those inhibitors, GDC0941 (Fig. 4a), BEZ235 (Fig. 4b), GSK 690693 (Fig. 4c), LY294002 (Fig. 4d) and CAL 101 (Fig. 4e) showed inhibitory effects on CEACAM1-4L, -4S, -3L, 3S mRNA levels, as compared with cells stimulated with IFN-γ and treated with vehicle, while levels of those mRNA expression were still higher than in cells stimulated and treated with vehicle (p < 0.05 or less, respectively). BEZ235 showed most strong inhibitory effect on both –S and –L isoforms. Levels of CEACAM1-4L and -3L were higher in cells treated with SHBM1009, while CEACAM-4S and -3S were lower in cells with SHBM1009 at high dose (Fig. 4f, p < 0.05 or less, respectively). Levels of CEACAM1 protein increased significantly 24 h after IFNγ stimulation (p < 0.01, vs control, Fig. 4g), which was down-regulated by treatment with GDC0941, GSK690693, BEZ235, LY294002, CAL101, or SHBM1009 (p < 0.01, vs IFNγ with vehicle, respectively). Levels of CEACAM1 protein in cells stimulated with IFNγ and treated with various PI3K inhibitors were still significantly higher than in those stimulated and treated with vehicle (p < 0.01, respectively, Fig. 5a).

Figure 5b demonstrated that levels of phosphatized Atk were lower in cell^CEACAM−^, and decreased even more significantly 10 min after the culture (p < 0.05, vs cell^CEACAM−^ at 0 min or cell^CEACAM+^, respectively).

## Discussion

CEACAM1 is a member of CECAM family in various cells derived from normal tissues or malignant tumors. CEACAM1 primary transcript generates 12 isoforms in human through alternative splicing, which are mainly allocated to long (L) and short (S) tails of intracellular domains. L tails contain two immunoreceptor tyrosine-based inhibitory motifs (ITIMs), coordinating inhibitory signaling, which S tails lack. CEACAM1-L mediates negative signaling, rather than CEACAM1-S [20]. CEACAM1 was involved in the processes of inflammation or tumor genesis, during which levels of CEACAM1 isoforms, or CEACAM1-S/CEACAM1-L changed in the pathological conditions. The present study found that the expression level of CEACAM1 was upregulated in AECOPD patients, and CEACAM1 could increase production of IL-6 and IL-8, which induced inflammation in the bronchial epithelial cells. CEACAM1 could regulate LPS driven production of IL-6 and IL-1β, mediating the appearance of clinical phenomes, e.g. fever response, through the ITIM receptor [21].

CEACAM1 as an important factor in carcinogenesis has dual roles of different isoforms [22]. For example, CEACAM-S isoform and CEACAM1-S/CEACAM1-L ratio were upregulated in non-small-cell lung cancer tissues [23], while CEACAM1-L isoforms in melanoma were upregulated [24]. Although the function of CEACAM1-L and CEACAM1-S in carcinogenesis seems varied among diseases, there is no doubt that CEACAM1 and its isoforms ratio is pivotal in carcinogenesis. The association between lung cancer and COPD has been emphasized for a while, even though the mechanism remains unclear [25]. The chronic inflammation, genetic susceptibility, DNA damage and repair are critical factors responsible for the occurrence of carcinogenesis [26–29]. Our data demonstrated that CEACAM1 was closely related to the severity of inflammation, cellular repair and migration which are critical in carcinogenesis. It indicates that CEACAM1 may be a key factor linking COPD and lung cancer, and the regulation of CEACAM1-S and -L isoforms may be a therapeutic target for COPD-lung cancer transit.

IFN-γ as a cytokine with a crucial immunological function against viral infections and mycobacteria, has also been suggested for treatment of malignant tumors [30], while recent study demonstrated that IFN-γ could promote cell malignant growth and even carcinogenesis of cancer after long-term application of IFN-γ [19], although the exact mechanism remains unclear. IFN-γ up regulates CEACAM1 expression in various epithelial cells, including human normal bronchial epithelial cells [31, 32]. The present study demonstrated that IFN-γ could stimulate airway epithelial cell proliferation and induce up-regulation of CEACAM1 expression, mainly CEACAM1-S isoforms.

**Fig. 6:**
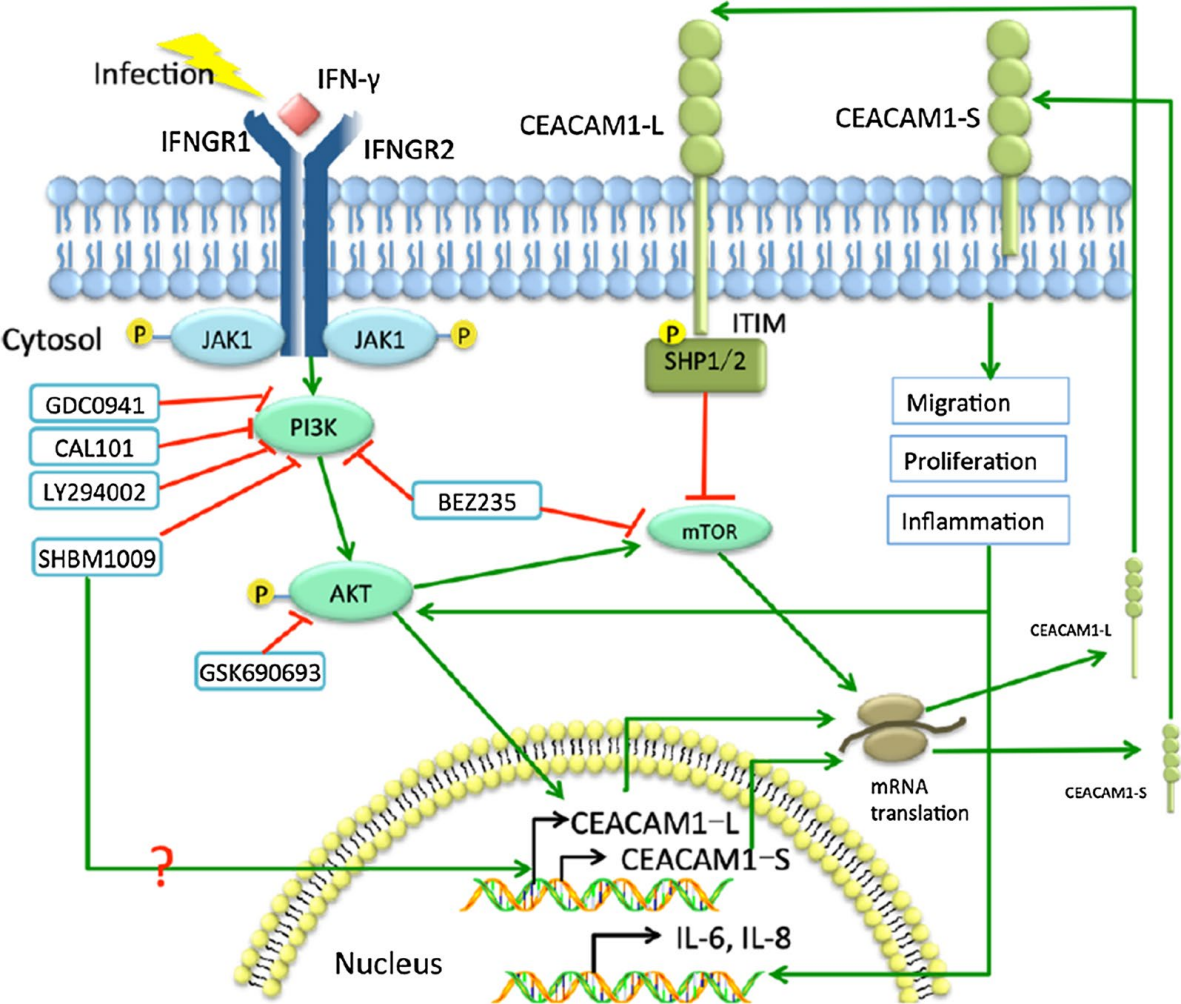
Potential mechanism of CEACAM1 and PI3K interactions. In the infectious environment during acute exacerbation of COPD, the increased IFN-γ up-regulated the expression of CEACAM1, especially the -S isoforms, which further promotes cellular migration, proliferation and induces the production of inflammatory factors such as IL-6 and IL-8. The -L isoforms of CEACAM1 contains two ITIMs in the cytoplasmic tail, which negatively regulates down-stream signal transduction pathways. PI3K/Akt pathway inhibitors (GDC0941, CAL101, LY294002, SHBM1009, BEZ235 and GSK69063) inhibited CAECAM1 expression, which suggested that PI3K/Akt pathway induces the up-regulation of CEACAM1 after IFN-γ stimulation. Different from other PI3K/Akt pathway inhibitors, SHBM1009 could specifically up regulate CEACAM1-L isoforms. As a positive feedback, CEACAM1 promoted the activation of PI3K/Akt pathway

PI3K plays an important role in cell growth, proliferation, differentiation, apoptosis, cytoskeleton, and intracellular trafficking through the function of four classes (Class I, II, III and IV) [33, 34]. In the present study, we chose 6 different PI3K inhibitors targeting various subunits of PI3K and investigated effects of those PI3K inhibitors on the signaling of downstream molecules (e.g. PI3Kp110α/δ, Akt, p110α/γ/δ/β/mTOR, PI3Kp110α/δ/β, PI3Kp110δ, or pan-PI3K), in order to understand the function of specific PI3K subunits on CEACAM1 and its isoforms’ expression. The present study showed that different kinds of PI3K pathway inhibitors could suppress the expression of CEACAM1 to various degrees, especially the CEACAM1-S isoforms. Our data indicate that Akt, PI3K/mTOR, and p110δ subunit are more involved in the regulation of CEACAM1-S and CEACAM1-L isoform functions, while p110α/δ and p110α/δ/β were much weaker. Comparing p110α/δ and p110α/δ/β, the p110δ subunit may play a more important role in IFN-γ-upregulated CEACAM1 expression. PI3K(p110δ)/Akt/mTOR may contribute more to the regulation of signaling pathway between IFN-γ and CEACAM1-S or CEACAM1-L isoforms in airway epithelial cells, as explained in Fig. 6. Our data firstly described that SHBM1009 up-regulated function of CEACAM1-L, while down-regulated CEACAM1-S, on basis of previous study [35]. However, the detailed target of SHBM1009 is unclear and the mechanism by which SHBM1009 regulates CEACAM1 isoforms needs to be further investigated. On the other hand, we also noticed that CEACAM1 also plays critical roles in IFN-γ-induced alterations of PI3K subunit expression and functions. The present study firstly explores the existence of intercommunication between CEACAM1 and PI3K at isoform/subunit levels, although such trade-off needs to be defined and validated in more precise targeting ways and in the in vivo system.

Our data evidenced that CEACAM1 plays a decisive role in inflammatory responses of epithelial cells through the production of inflammatory mediators, which is at least one of critical effects. We also found IFN-γ induced the overproduction of IL-6 and IL-8 through CEACAM1, which may take part in the pathogenesis of AECOPD or the transition from inflammation to cancer [36, 37]. We furthermore found that CEACAM1 promoted the migration and regeneration of airway epithelial cells, which promoted the malignancy in tumors. Studies on malignant tumors showed that the effect of CEACAM1 on cellular proliferation depends on the ratio of L/S isoforms, e.g. CEACAM1-S promotes proliferation while CEACAM-L has a totally opposite effect [38]. Our results suggested that CEACAM1-4S and CEACAM1-3S were more involved in IFN-γ-induced epithelial cell proliferation and migration, and might be the promoters to bridge between chronic inflammations and cancer.

## Conclusion

In conclusion, IFN-γ up-regulated the expression of CEACAM1 in airway epithelial cells, especially CEACAM1-S isoforms, promoting cellular proliferation, migration, and the production of inflammatory factors. PI3K (p110δ)/Akt/mTOR pathway was involved in the process of IFN-γ-upregulated CEACAM1, especially CEACAM1-S. On the other hand, CEACAM1 could promote the activation of PI3K/Akt/mTOR pathway. Thus, we believe that the signal pathway between CEACAM1-S and PI3K (p110δ)/Akt/mTOR can be new alternative of future therapies against epithelial transition from inflammation to cancer.

## Additional file

**Additional file 1: Figure S1.** Expression of CEACAM1 after LPS stimulation in HBE cells. CEACAM1 gene expression in HBE cells treated with LPS at concentration of 0, 0.1, 1 μg/ml for 24 h, no significant change of CEACAM1 was shown.

## Abbreviations

CEACAM1: carcinoembryonic antigen-related cell adhesion molecule 1
AECOPD: acute exacerbation of chronic obstructive pulmonary disease
IFN-γ: interferon-γ
IL-6: interleukin 6
IL-8: interleukin 8
CCK-8: cell counting kit-8
PI3K: phosphatidylinositide 3-kinase
COPD: chronic obstructive pulmonary disease
PBMC: peripheral blood mononuclear cell
LPS: lipopolysaccharide
siRNA: small interfering RNA
TGF-β: transforming growth factor-β
VEGF: vascular endothelial growth factor
MCP-1: monocyte chemoattractant protein-1
