# Supplementary figures and images for: Interferon gamma induces inflammatory responses through the interaction of CEACAM1 and PI3K in airway epithelial cells
Source: J Transl Med. 2019 May 9;17:147. doi: 10.1186/s12967-019-1894-3 (PMC6507156; doi:10.1186/s12967-019-1894-3)

Additional figure S1


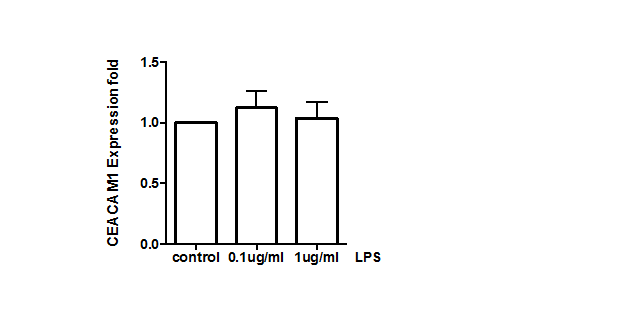

Supplement: Supplementary file 1 — Additional file 1: Figure S1. Expression of CEACAM1 after LPS stimulation in HBE cells. CEACAM1 gene expression in HBE cells treated with LPS at concentration of 0, 0.1, 1 μg/ml for 24 h, no significant change of CEACAM1 was shown. [file 12967_2019_1894_MOESM1_ESM.docx]
